# Underwood Spring Water

**Published:** 1886-03

**Authors:** 


### UNDERWOOD SPRING WATER.

For some time we have been using Underwood Spring Water for the table, and in cases of urinary derangements. As a beverage it is superior to either Apollinaris or Congress. As a diuretic it is better than many of the numerous waters which have hitherto been recommended, because it is more mild. In its action upon the digestive apparatus it is less drastic than Hunyadi, while it is exceedingly palatable. As a diluent for Claret, when such is desired, it acts admirably as a corrective for the astringent effect upon some people. Altogether, it is the most agreeable table water that we have ever used.

